# Evolution and prediction of mismatch between observed and perceived upper limb function after stroke: a prospective, longitudinal, observational cohort study

**DOI:** 10.1186/s12883-021-02493-1

**Published:** 2021-12-15

**Authors:** Bea Essers, Annick Van Gils, Christophe Lafosse, Marc Michielsen, Hilde Beyens, Fabienne Schillebeeckx, Janne M. Veerbeek, Andreas R. Luft, Daphne Kos, Geert Verheyden

**Affiliations:** 1 Department of Rehabilitation Sciences, KU Leuven, Tervuursevest 101 box 1501, 3001 Leuven, Belgium; 2 Department of Allied Health and Department of Research, Rehabilitation Hospital RevArte, Antwerp, Belgium; 3 Rehabilitation Campus Sint Ursula, Jessa Hospital, Hasselt, Belgium; 4 Department of Physical Medicine and Rehabilitation, University Hospitals Leuven, Leuven, Belgium; 5 Luzerner Kantonsspital, Neurocenter, Lucerne, Switzerland; 6 Division of Vascular Neurology and Neurorehabilitation, Department of Neurology, University of Zurich and University Hospital Zurich, Zurich, Switzerland; 7 Cereneo, Center for Neurology and Rehabilitation, Vitznau, Switzerland

**Keywords:** Stroke, Upper limb, Outcome

## Abstract

**Background:**

A previously shown ‘mismatch’ group of patients with good observed upper limb (UL) motor function but low perceived UL activity at six months post stroke tends to use the affected UL less in daily life than would be expected based on clinical tests, and this mismatch may also be present at 12 months. We aimed to confirm this group in another cohort, to investigate the evolution of this group from six to 12 months, and to determine factors on admission to inpatient rehabilitation and at 6 months that can discriminate between mismatch and good match groups at 12 months.

**Methods:**

Persons after stroke were recruited on rehabilitation admission and re-assessed at six and 12 months. Observed UL function was measured with the upper extremity subscale of the Fugl-Meyer Assessment (FMA-UE) and perceived UL activity by the hand subscale of the Stroke Impact Scale 3.0 (SIS-Hand). We defined mismatch as good observed UL function (FMA-UE > 50/66) but low perceived activity (SIS-Hand≤75/100). Potential discriminators at admission and 6 months (demographic characteristics, stroke characteristics, UL somatosensory function, cognitive deficits, mental function and activity) were statistically compared for match and mismatch groups at 12 months.

**Results:**

We included 60 participants (female: 42%) with mean (SD) age of 65 (12) years. We confirmed a mismatch group of 11 (18%) patients at 6 months, which increased to 14 (23%) patients at 12 months. In the mismatch group compared to the good match group at 12 months, patients had a higher stroke severity and more somatosensory impairments on admission and at 6 months.

**Conclusions:**

We confirmed a group of patients with good observed UL function but low perceived activity both at six and at 12 months post stroke. Assessment of stroke severity and somatosensory impairments on admission into rehabilitation could determine mismatch at 12 months and might warrant intervention. However, large differences in clinical outcomes between patients in the mismatch group indicate the importance of tailoring training to the individual needs.

## Background

Upper limb (UL) motor function is impaired in the majority of patients after stroke [1], leading to difficulties in using the UL in daily life and performing daily activities [2]. These difficulties remain often present in the chronic phase after stroke (after 6 months [3]) and can lead to a lower level of independence [4] and quality of life [5]. In order to reduce impaired UL functioning and its consequences, it is important to assess UL functioning.

UL functioning, as defined by the World Health Organization (WHO) in the International Classification of Functioning, Disability and Health (ICF) [6], is an umbrella term for all UL functions, activities and participation. UL functions can be assessed with observation-based assessments, whereby the therapist observes and scores the patient’s function of the UL. Next to these observation-based assessments, patient-reported outcomes might add valuable information. Patient-reported outcomes for the UL reflect how a person subjectively experiences the level of UL activity in the current environment: the perceived UL activity [7].

Despite a strong correlation between this perceived UL activity and the observed UL function, these measurements are not always congruent. A considerable group of patients in the acute and subacute phase post stroke (range: 20–71%) demonstrated a good observed UL function, but a lower perceived UL activity, this is referred to as the mismatch group [8–12]. At the beginning of the chronic phase post stroke, this mismatch group was still present in one of our earlier studies: in a group of 32 stroke patients at six months post stroke, one in five showed a good observed UL function but low perceived UL activity [13]. This mismatch between good observed UL function but low perceived UL activity might have implications for the actual daily life UL activity, or how (much) a patient uses the UL in daily life [14]. A pre-requisite for a good daily life UL activity is having a good observed UL function [15–17], which is fulfilled in the mismatch group. However, this might not be enough. Patients with good UL function show a wide variation in daily life UL activity [18, 19] and improved UL function does not always translate into improved daily life UL activity [20]. This variation in daily life UL activity may be linked to the perceived UL activity. A lower perception of confidence in one’s UL activity was shown to affect impaired UL use during early stroke rehabilitation, with higher confidence related to greater paretic UL use [21, 22]. Also in the chronic phase post stroke, lower confidence in one’s UL activity was associated with higher non-use of the impaired UL in daily life [23]. A recent cross-sectional study in the chronic phase post stroke investigated the actual daily life UL activity in two groups of patients with similar good observed UL activity, but a different perceived UL activity [24]. We found that, compared to patients with good perceived UL activity (good match group), patients with a low perceived UL activity (mismatch group) tend to use their affected UL less in daily life [24]. To conclude, despite a good observed UL function, patients in this mismatch group might be at risk for reduced UL activity in daily life.

As the perceived UL activity tends to plateau (62%) or even decreases (14%) from 3 to 12 months post stroke [25] and deteriorates further up to 6 years post stroke [26], we expect that the mismatch group will increase along the chronic phase post stroke. However, if we could determine factors in the (sub)acute and start of the chronic phase that distinguish the mismatch group from the good match group, therapists could incorporate these factors in therapy and try to prevent patients from developing the mismatch. As the mismatch group differs from the good match group in perceived UL activity, factors related to a low perceived UL activity might also differentiate the mismatch from the good match group. Several factors are related to a low self-perceived UL activity after stroke, including lower cognition [27], lower age, higher stroke severity, lower somatosensory function and lower independence in activities of daily living (ADL) at rehabilitation discharge [28] and a higher level of education and a lower mood score in the subacute phase post stroke [8].

The aim of this longitudinal prognostic study is threefold. First, we aim to confirm the match and mismatch groups in a larger, new cohort at six months post stroke. Second, we want to investigate the evolution of the mismatch group from six to 12 months post stroke. Third, we want to determine factors on admission to inpatient rehabilitation and at six months post stroke that can discriminate between the mismatch and good match group at 12 months post stroke. We hypothesized that the number of patients in the mismatch group would increase from six to 12 months [16, 17]. We further assumed that stroke severity on admission, age, somatosensory function, ADL independence, education and mood can distinguish between mismatch and good match at 12 months [8, 27, 28].

## Methods

### Design and setting

This was a retrospective secondary analysis from a prospective longitudinal cohort study investigating predictors of bimanual performance within the first year after stroke [29]. Patients post stroke were recruited from three rehabilitation centres in Belgium (University Hospitals Leuven, Jessa Hospital Rehabilitation Campus Sint Ursula Herk-De-Stad and Rehabilitation Hospital RevArte Edegem) between February 2016 and November 2017 and assessed by one trained researcher (AVG).

### Participants

Participants were included within 1 week after admission to the rehabilitation centre if they met the following inclusion criteria 1) first-ever unilateral, supratentorial stroke, as defined by the American Heart Association/American Stroke Association [30], 2) unilateral motor deficit in the UL (upper extremity subscale of the Fugl-Meyer Assessment (FMA-UE) < 60/66) [31], 3) age 18 years or older, and 4) admission to the rehabilitation centre within 6 weeks after stroke onset. Patients were excluded if they had 1) other neurological diseases, such as multiple sclerosis or Parkinson’s disease, 2) stroke-like symptoms caused by subdural hematoma, tumour, encephalitis or trauma, 3) serious cognitive or communication deficits restricting the evaluation and 4) no written informed consent. As this study was exploratory, and there is limited information on the predictors for mismatch, a sample size calculation was not possible. Ethical approval for secondary data analysis was granted by the Ethics Research Committee of UZ/KU Leuven (S58670). All procedures followed were in accordance with the Declaration of Helsinki. Our study conformed to STROBE Statement [32].

### Outcome measures

At six and 12 months, observed function of the affected UL was assessed with the reliable and valid FMA-UE, with a total score between 0 and 66 and higher scores indicating better observed function [31, 33]. Perceived UL activity was evaluated using the hand subscale of the reliable and valid Stroke Impact Scale 3.0 (SIS-Hand), with a total score between 0 and 100 and higher scores indicating better perceived function [34, 35].

On admission to inpatient rehabilitation, we collected 1) patient-related, demographic characteristics including age at stroke onset, gender, pre-stroke residential status and pre-stroke educational level, 2) stroke characteristics including days since stroke onset, stroke aetiology, lateralization of stroke [36], 3) stroke severity (National Institutes of Health Stroke Scale) [37] and 4) clinical characteristics. These clinical characteristics included UL somatosensory function by means of the Erasmus MC modifications of the revised Nottingham Sensory Assessment (Em-NSA) for exteroception and proprioception [38, 39] and stereognosis using the subscale of the original Nottingham Sensory Assessment [39], cognitive deficits by the Montreal Cognitive Assessment (MoCA) [40, 41], mental function with the Hospital Anxiety and Depression Scale (HADS) [42]; and level of independence in activities of daily living with the 100-point Barthel Index (BI) [43]. Clinical characteristics were re-assessed at six- and 12-months post stroke.

### Data analysis

Patients’ characteristics are presented as mean with standard deviation (SD) (continuous variables), median with first quartile (Q1) and third quartile (Q3) when continuous variables were not normally distributed (Shapiro Wilk test and histogram) and frequency with percentage (categorical variables). To discriminate between low and good function, we used cut-off scores based on previous literature [24, 44–48]. For the FMA-UE, 50/66 discriminated between low (≤50) and good (> 50) observed function, and 75/100 on the SIS-Hand distinguished between low (≤75) and good (> 75) perceived activity. We then identified three groups at six and 12 months: 1) patients with low observed function and low perceived activity (low match group), 2) patients with good observed function and good perceived activity (good match group), and 3) patients with good observed function but low perceived activity (mismatch group). For evolution, we report change in number of patients in the mismatch group from six to 12 months and we check whether patients in the mismatch group at six months stay in this subgroup at 12 months.

As we also wanted to investigate what distinguishes low from good perceived function among those individuals with a good observed function, we focused on the distinction between the mismatch and good match group. To examine differences in potential discriminating factors on admission to inpatient rehabilitation and at six months for group membership at 12 months, we used Chi-square tests for categorical variables and independent t-tests or Mann-Whitney U tests for normally and not-normally distributed continuous variables. Data were analysed with the IBM SPSS Statistics version 27 (IBM Corporation, Armonk, NY, USA) with the level of statistical significance set at *p* < 0.05 (2-sided).

## Results

We included patients with FMA-UE and SIS-Hand values at both six and 12 months, which resulted in a sample of 60 out of 68 patients included at 6 months. At 12 months, four were medically unstable, three declined to take part in the assessment and one started participation in another study. Patients with incomplete data at 12 months (*n* = 8) were not different from the patients with complete data at 12 months (*n* = 60) for scores on Em-NSA Exteroception (*p* = 0.92), Em-NSA Proprioception (*p* = 0.76), NSA Stereognosis (*p* = 0.63), MoCA (*p* = 0.55), HADS total (*p* = 0.79), and Barthel index (*p* = 0.70) at six months. Patients in our sample had a mean age of 65 (SD = 12) years and were recruited at a mean of 22 (SD = 8) days post stroke. Out of 60 participants, 58% (*n* = 35) were male, 62% (*n* = 37) had a right-sided hemiparesis and 87% (*n* = 52) suffered an ischemic stroke. Overall, observed UL function as assessed by the FMA-UE on admission was severely to moderately impaired with a median score of 10 out of 66 (Q1-Q3 = 5–44), moderately impaired at six months (median = 47, Q1-Q3 = 14–58) and mildly impaired at 12 months post stroke (median = 50, Q1-Q3 = 19–61). Perceived UL activity was not assessed on admission, but was overall low at six months with a median score of 50 out of 100 (Q1-Q3 = 5–80) and decreased to a median score of 45 (Q1-Q3 = 0–79) at 12 months.

### Evolution of the mismatch group

**Fig. 1:**
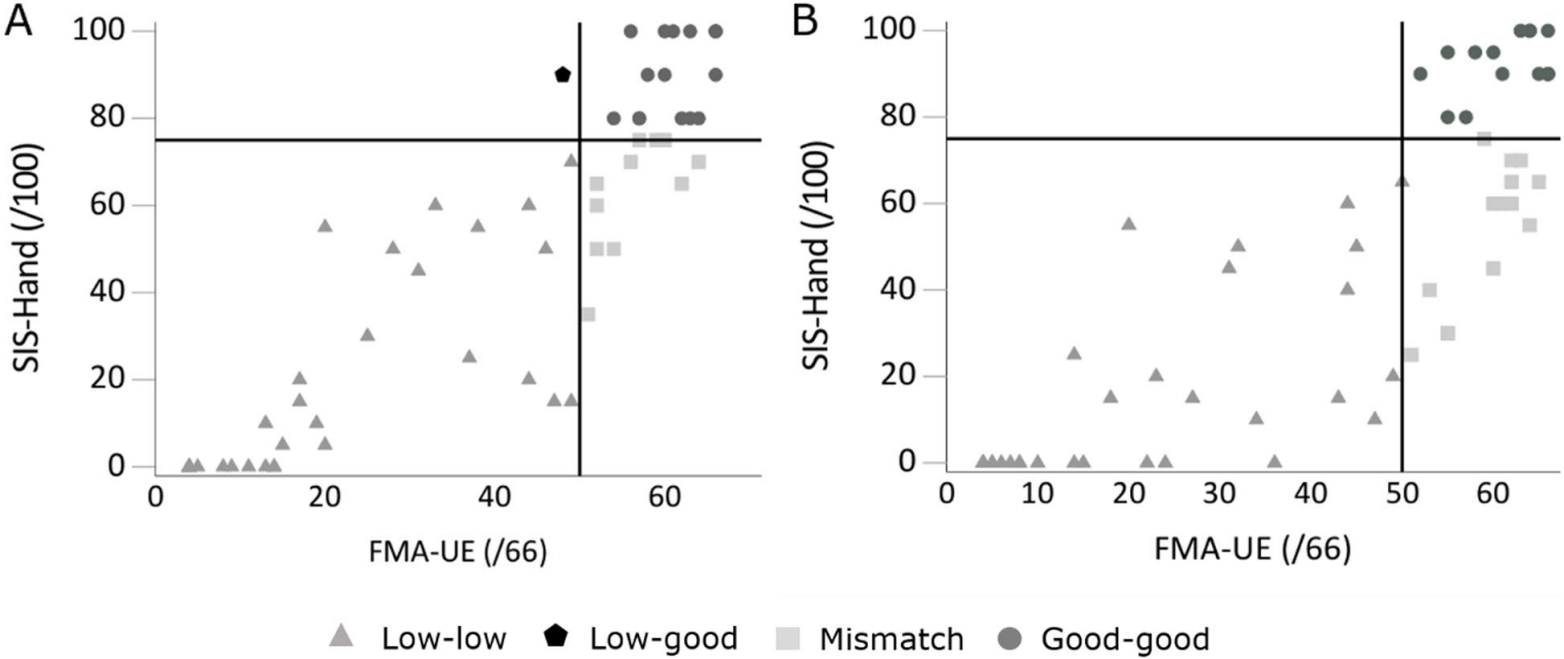
Scatterplot of observed upper limb function (FMA-UE) and perceived upper limb activity (SIS-Hand) at six and 12 months

Figure 1A presents the scatterplot between observed function and perceived activity at six months. We identified patients with low observed function and low perceived activity (low match group; *n* = 33; 55%), a group with good observed function and good perceived activity (good match group; *n* = 15; 25%) and a group with good observed function but low perceived activity (mismatch group; *n* = 11; 18%). At 12 months (Fig. 1B), the number of patients in the low match group decreased (*n* = 31; 52%), remained unchanged in the good match group and increased in the mismatch group (*n* = 14; 23%).

Observed function (Fugl-Meyer Assessment upper extremity; FMA-UE, range 0–66) versus perceived activity (hand subscale of the Stroke Impact Scale; SIS-Hand, range 0–100) at **A** six and **B** 12 months post stroke. From six to 12 months, the number of patients with a mismatch increased from 11 (18%) to 14 (23%) patients.

The majority of the patients in the mismatch group at six months stayed in this mismatch group at 12 months (*n* = 9/11; 82%). The other two patients in the mismatch group at six months moved to the good match group at 12 months, as they increased their perceived UL activity, which was accompanied by an increase in FMA-UE score of three and six points. At 12 months post stroke, almost half of the patients with good observed UL function had a low perceived UL activity (*n* = 14/29). Five people entered the mismatch group, coming from the low match group (n = 1, 20%) and the good match group (*n* = 4, 80%). For those entering the mismatch group from the good match group, the FMA-UE score did not change or improved only slightly with two points, whereas the SIS-Hand score decreased. At six months post stroke, three patients had a SIS-Hand score of 80, which decreased with five, ten and 25 points up to 12 months post stroke. The fourth patient started with a SIS-Hand score of 90 at six months post stroke, which decreased to 60 at 12 months.

### Discriminative factors

Admission characteristics and comparison of potential discriminative factors on admission to rehabilitation for group membership at 12 months are provided in Table 1.

**Table 1:** Comparison of demographic and stroke-related characteristics on rehab admission for mismatch and good match groups at 12 months, n (%) and median (Q1-Q3)

Variable	*P*-value	Mismatch (*n* = 14)	Good match (*n* = 15)
*Demographics*			
Age at stroke onset, mean (SD) ^a^	0.882	65 (14)	65 (14)
Gender, male	0.876	8 (57)	9 (60)
Pre-stroke residential status, living together	0.897	8 (57)	8 (53)
Educational level	0.760		
Primary education		0 (0)	1 (7)
Lower secondary education		5 (36)	5 (33)
Higher secondary education		5 (36)	3 (20)
Higher tertiary education		4 (28)	6 (40)
*Stroke-related characteristics*			
Days since stroke onset, mean (SD) ^a^	0.716	20 (7)	18 (7)
Type of stroke, ischemic	0.564	11 (79)	13 (87)
Stroke lateralization, right hemiparesis	0.742	11 (79)	11 (73)
Affected upper limb = pre-stroke dominant	0.060	12 (86)	8 (53)
Stroke severity (NIHSS/42)	0.029*	6 (4–9)	4 (2–6)

All between group comparisons were performed with Mann-Whitney U test, except for ^a^comparison with independent t-test

*NIHSS* National Institutes of Health Stroke Scale, *SD* Standard Deviation

*Significant at *p* < 0.05

Demographic and stroke-related characteristics did not differ between groups, except for a higher initial stroke severity in the mismatch group (median NIHSS = 6, Q1-Q3 = 4–9) compared to the good match group (median NIHSS = 4, Q1-Q3 = 2–6; *p* = 0.029). For the demographic data, mean age in both groups was 65 (SD = 14) years, the majority was male (57% in the mismatch and 60% in the good match group), about half of the participants was living together (57% in mismatch and 53% in good match group) and all but one participant followed lower secondary education or more. For the stroke-related characteristics, participants were on average 20 (mismatch) and 18 days (good match) post stroke and most participants had an ischemic stroke (79 and 87%) with a right hemiparesis (79 and 73%). Although more people in the mismatch group had the dominant hand affected compared to the good match group (86% versus 53%), this difference was not significant (*p* > 0.05).

Table 2 provides the comparison of clinical characteristics at baseline and six months post stroke for group membership at 12 months. At baseline, Em-NSA Exteroception scores were higher in the good match group (median = 31, Q1-Q3 = 28–32) compared to the mismatch group (median = 22, Q1-Q3 = 12–32; *p* = 0.006) as well as Em-NSA Proprioception scores (median = 8, Q1-Q3 = 8–8 versus median = 7, Q1-Q3 = 4–8; *p* = 0.041). At 12 months post stroke, Em-NSA Exteroception scores were still higher in the good match group (median = 32, Q1-Q3 = 30–32) compared to the mismatch group (median = 30, Q1-Q3 = 26–31; *p* = 0.037). Last, also NSA Stereognosis scores at 12 months were higher in the good match group (median = 20, Q1-Q3 = 19–21) compared to the mismatch group (median = 19, Q1-Q3 = 11–20; *p* = 0.012).

**Table 2:** Comparison of clinical characteristics on rehab admission and at 6 months for mismatch and good match groups at 12 months, n (%) and median (Q1-Q3)

Variable	*P*-value	Mismatch (n = 14)	Range	Good match (n = 15)	Range
*Rehab admission*					
Em-NSA Exteroception (/32)	0.006**	22 (12–30)	0–32	31 (28–32)	20–32
Em-NSA Proprioception (/8)	0.041*	7 (4–8)	0–8	8 (8–8)	6–8
NSA Stereognosis (/22)	0.130	6 (1–20)	0–22	18 (15–19)	0–22
Cognition (MoCA/30)	0.052	21 (16–24)	6–28	25 (22–27)	13–29
HADS total score (/42) (*n* = 28)	0.548	11 (6–16)	1–18	9 (5–15)	0–21
Barthel Index (/100)	0.217	48 (30–86)	20–100	75 (55–85)	30–100
*Six months*					
Em-NSA Exteroception (/32)	0.037*	30 (26–31)	0–32	32 (30–32)	29–32
Em-NSA Proprioception (/8)	0.331	8 (8–8)	4–8	8 (8)	8–8
NSA Stereognosis (/22)	0.012*	19 (11–20)	0–21	20 (19–21)	18–22
Cognition (MoCA/30)	0.063	24 (22–26)	10–28	26 (24–28)	20–30
HADS total score (/42)	0.080	9 (7–16)	1–21	7 (1–9)	0–18
Barthel Index (/100)	0.063	98 (90–100)	45–100	100 (100–100)	95–100

All between group comparisons were performed with Mann-Whitney U test

*Em-NSA* Erasmus MC Modifications of the revised Nottingham Sensory Assessment, *HADS* Hospital Anxiety and Depression Scale, *MoCA* Montreal Cognitive Assessment; NSA: Nottingham Sensory Assessment

*significant at *p* < 0.05

** Significant at *p* < 0.01

**Fig. 2:**
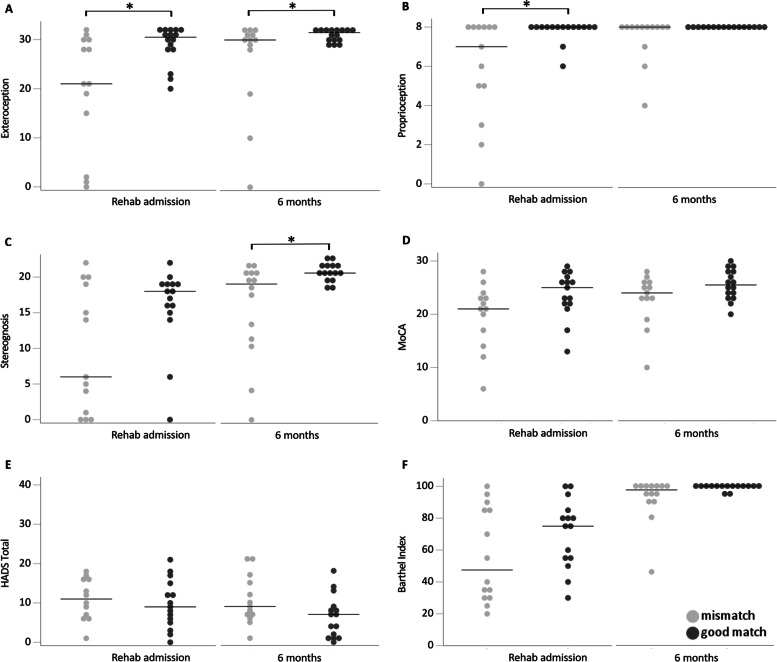
Scatterplot of clinical variables on admission to rehabilitation and six months post stroke for the mismatch group compared to the good match group at 12 months

Figure 2 shows the evolution of discriminating factors over time. Looking at the median values (horizontal bar) for both groups, all values improve from admission to 6 months post stroke. The median Em-NSA Proprioception value for the good match group was already maximal on admission and could thus not further improve up to 6 months post stroke. For the other variables, improvements in scores go along with a decrease in score ranges for the good match group, but not for the mismatch group. The large score range for the mismatch group indicates high variation in scores between the individuals in this group.

Scatterplot of clinical variables for the mismatch group (*n* = 14; FMA-UE > 50, SIS-Hand ≤75) and good match group (*n* = 15; FMA-UE > 50, SIS-Hand > 75) on admission and at six months post stroke. Every dot represents the raw value of a patient; raw median scores are indicated with a horizontal bar. Horizontal brackets with an asterisk indicate significant differences between two groups (*p* < 0.05). **A** Em-NSA Exteroception (Erasmus modified Nottingham Sensory Assessment Exteroception /32), **B** Em-NSA Proprioception (/8), **C**. NSA Stereognosis (Nottingham Sensory Assessment Stereognosis /22), **D** MoCA (Montreal Cognitive Assessment /30), **E** HADS Total (Hospital Anxiety and Depression Scale /42), and **F** Barthel Index (/100).

## Discussion

This study hypothesized that the number of patients in the mismatch group would increase from six to 12 months post stroke. We further assumed that stroke severity at rehab admission, age, somatosensory function, ADL independence, education and mood could distinguish between mismatch and good match at 12 months. The current findings in large part support the hypotheses, as we confirmed a mismatch group as six- and 12-months post stroke, with the latter being relatively greater. We also demonstrated that people in the mismatch group had a higher stroke severity and somatosensory impairment on admission to the rehabilitation centre and at six months post stroke compared to persons in the good match group.

The mismatch group of individuals with good observed function and low perceived UL activity we found at six- and 12-months post stroke, was described in other studies in the earlier rehabilitation phase. Two of these studies described an additional mismatch group of patients with low observed and high perceived functional improvement [8, 12]. This was also shown for one person in our sample at six months post stroke. At 12 months post stroke however, we did not find such group, which can be explained by a difference in time frame: in the early rehabilitation phase, individuals may have lower insight in and overestimate their capabilities. In the later phase post stroke, one study showed 19% of patients had a mismatch at six months [13], which is very similar to the 18% at six months we found. This is less than the 11 out of 43 (26%) individuals reporting difficulty with hand movements despite normal examination in another study [11], which can be explained by several factors. Results were present over a wide range of times post stroke, individuals had an overall moderate motor impairment and good observed function and perceived activity were defined as maximum score on FMA-UE and SIS-Hand, which in our opinion is a too strict criterion.

When interpreting the evolution of the mismatch prevalence from six to 12 months, we see that the mismatch group remains important throughout the chronic phase post stroke. The majority of patients in the mismatch group at six months stayed in the mismatch group at 12 months. From six to 12 months, only two patients moved from the mismatch group to the good match group, accompanied by an improvement in the observed UL motor function. Twice as many patients moved from the good match to the mismatch group, accompanied by a decrease in perceived UL activity. Three of these four patients had a perceived UL activity just above the cut-off value and were thus borderline in the good match group. The fourth patient had a clearly distinguishable good perceived UL activity at six months, which decreased with 30% by twelve months. This large decrease might be explained by the patient’s higher depression and anxiety values, lower cognition and lower somatosensory function at six months post stroke compared to the good match sample who did not change group.

All patients who switched from the good match to the mismatch group had a lower exteroceptive function at admission to rehabilitation compared to the good match sample who did not change group, except for one. This participant had higher depression and anxiety values on admission to rehabilitation compared to the non-switching good match subgroup. To summarize, despite having a good observed UL function and good perceived UL activity at six months post stroke, patients with low exteroceptive function or high depression and anxiety at admission to rehabilitation might be at risk for developing a mismatch at 12 months post stroke. Furthermore, even in the absence of somatosensory deficits, patients with low cognitive function or high depression and anxiety values at six months might be at risk for developing the mismatch at 12 months post stroke.

Discriminating factors on admission for mismatch at 12 months were higher stroke severity and more somatosensory impairments. Stroke severity is one of the most powerful predictors of stroke recovery [49] and a higher stroke severity at admission to rehabilitation was associated with lower perceived UL activity scores [11] in the subacute phase post stroke. It was previously shown that initial somatosensory impairment is significantly related to somatosensory ability at six months [50] and a similar trend was seen in our sample. Although somatosensory function improved from admission to six months in the mismatch as well as the good match group, it remains lower in the mismatch group compared to the good match group at six months post stroke. It was shown earlier that somatosensory function plays an important role in the perceived UL activity in chronic stroke patients with lower observed UL motor function [51]. Based on our findings, it might thus also play a discriminative role in chronic stroke patients with good observed UL motor function for their perceived UL activity.

As somatosensory function on admission seems to be an important discriminator between good match and mismatch at 12 months post stroke, we might want to include somatosensory retraining in an early stage in order to improve the perceived UL activity in our chronic mismatch group. However, the evidence for somatosensory retraining in the early stage post stroke is limited and mostly focused on improving UL motor function and activity [52]. When the goal is to improve UL motor function, a pure somatosensory approach is not enough [53] and is therefore often combined with motor training. Results of this sensorimotor therapy show that it might not improve somatosensory function and may be less effective than pure motor therapy for motor recovery in the early rehabilitation phase post stroke [54].

The mismatch group in our sample does not only have lower sensorimotor function than the good match group on admission, but also at six months post stroke. Further, patients moving from the good match group to the mismatch group from six to 12 months post stroke have on average a lower somatosensory function at six months. Therefore, it might be beneficial to include somatosensory retraining in the early chronic phase to prevent a (switch to) mismatch at 12 months. The majority of trials investigating the effect of sensorimotor training have focused on patients in the chronic phase post stroke [53]. Despite the potential to improve UL somatosensory and motor capacity, the evidence of efficacy of these sensorimotor interventions is limited [55]. This limited evidence suggests that sensory training may be effective as a supplemental training program and should be individually tailored [56].

To our knowledge, so far only one study investigated the effect of somatosensory retraining in the chronic phase post stroke on perceived UL activity [57]. Although the average perceived UL activity increased immediately after training and at follow-up, the perceived UL activity after retraining was highly variable among participants. This wide range in perceived UL activity changes could be associated to other influencing variables. In our sample, patients switching from the good match to the mismatch group had a more impaired cognition and higher depression and anxiety compared to patients who stayed in the mismatch group. It seems plausible that more depressed and anxious patients perceive a lower hand function and report more negative outcomes than those with a better mood [58]. Furthermore, it was shown that anxiety and depression may not only interfere with functional recovery [59], they also seem to reduce intention to perform a voluntary motor task [60]. In interpreting the findings of the present study, some limitations should be noted. First,although this study provided some preliminary evidence regarding factors on admission and at six months post stroke that may discriminate between mismatch and good match, the observational design of this study cannot provide evidence for causality. Further, because of the relatively small sample size and large score range of clinical outcomes, we should view the results of the study with caution. Prospective studies with larger sample sizes would be needed in order to evaluate how these discriminators could potentially predict the perceived upper limb activity in persons with good UL motor function in the chronic phase post stroke. We did not perform a predictive analysis as the number of participants with the outcome is important in prediction model studies, as it influences precision and affects predictive performance [61]. For model development studies, sample size considerations are often based on events per variable (EPV), in which prediction models based on studies with an EPV lower than 10 are likely to have overfitting (including spurious predictors) or underfitting (failing to include important predictors) [62, 63]. As we had only 14 participants with the outcome (mismatch) and eight potential predictors, the current sample was not appropriate to perform prediction analysis.

This study has some clinical implications. First, in the prevention of mismatch in the chronic phase post stroke, it might be important to distinguish participants with higher stroke severity and more severe somatosensory impairments on rehab admission and at 6 months post stroke, as these patients are at risk of developing a mismatch at 12 months. For this group of patients, it may be of benefit to include somatosensory retraining on admission to rehabilitation. However, given the inconclusive results of somatosensory retraining based on previous research [57] and the wide ranges in somatosensory scores in our mismatch group at 12 months, somatosensory retraining might not be beneficial for all individuals in the mismatch group. Second, despite improvement in somatosensory function, some patients move from the good match at six months post stroke to the mismatch group at 12 months. Therefore, it might be important to assess influencing factors such as high depression and anxiety and low cognition, which potentially induce a decrease in perceived UL activity throughout the chronic phase post stroke. Tailoring training to individual needs seems to be the key in this diverse mismatch group.

## Conclusions

In conclusion, a considerable group (~ 20%) of patients displays a good observed function but a low perceived UL activity not only at the start of, but also later in the chronic phase post stroke. Factors that discriminate between groups are higher stroke severity at admission to the rehabilitation centre and lower somatosensory function at six months in the mismatch group. Although training might include somatosensory retraining, large differences in clinical outcomes between patients in the mismatch group indicate that it is important to tailor training to the individual needs.

## Data Availability

The data analysed during the current study are available from the corresponding author, Bea Essers, on reasonable request.

## Abbreviations

BI: Barthel Index
Em-NSA: Erasmus MC modifications of the revised Nottingham Sensory Assessment
FMA-UE: Fugl-Meyer Assessment upper extremity
HADS: Hospital Anxiety and Depression Scale
MoCA: Montreal Cognitive Assessment
NIHSS: National Institutes of Health Stroke Scale
NSA: Nottingham Sensory Assessment
Q1: First quartile
Q3: Third quartile
SD: Standard deviation
SIS-Hand: Stroke Impact Scale version 3.0 hand subscale
UL: Upper limb
